# Theoretical model of impact mitigation mechanisms inherent to the North American bison skull

**DOI:** 10.1242/bio.060517

**Published:** 2024-09-19

**Authors:** Andrea Karen Persons, Youssef Hammi, Steven H. Elder, Lauren B. Priddy, Matthew W. Priddy, Ryan Butler, Avery Schemmel, Elizabeth Whitehurst, Nayeon Lee, Mark F. Horstemeyer

**Affiliations:** ^1^ Jameson Crane Sports Medicine Institute, The Ohio State Wexner Medical Center, Columbus, OH 43202, USA; ^2^ Center for Advanced Vehicular Systems, Mississippi State University, Starkville, MS 39759, USA; ^3^ Department of Biological and Agricultural Engineering, Mississippi State University, Mississippi State, MS 39762, USA; ^4^ Department of Mechanical Engineering, Mississippi State University, Mississippi State, MS 39762, USA; ^5^ Department of Veterinary Clinical Sciences College of Veterinary Medicine, The Ohio State University, Columbus, OH 43210, USA; ^6^ School of Engineering, Liberty University, Lynchburg, VA 24515, USA

**Keywords:** Bison, Reproduction, Cortical bone, Plexiform bone, Mechanical properties of bone, Finite element analysis

## Abstract

North American bison (Bovidae: *Bison bison*) incur blunt impacts to the interparietal and frontal bones when they engage in head-to-head fights. To investigate the impact mitigation of these bones, a finite element analysis (FEA) of the skull under loading conditions was performed. Based on anatomical and histological studies, the interparietal and frontal bones are both comprised of a combination of haversian and plexiform bone and are both underlain by bony septa. Additionally, the interparietal bone is thicker than the frontal bone. Data regarding the mechanical properties of bison bone are scarce, but the results of a phylogenetic analysis infer that the material properties of the closely related domestic cow bone are a suitable proxy for use in the FEA. Results of the FEA suggest that the thickness of the interparietal bone in conjunction with the bony septa may prevent fracture stresses by helping to absorb and disperse the blunt impact energy throughout the skull. Monotonic stress levels of 294 MPa, which are below the compressive strength of bone were exhibited in the simulated bison head impacts indicating no fracture of the bones.

## Teaser

**Summary:** The bony septae underlying the frontal and interparietal bones of the bison skull help to dissipate the impact energy produced when two bison engage in head-to-head fighting. Oblique impacts, whereby one bison uses its interparietal bone to hit another bison in the side of the head, produce the highest peak strains.

## INTRODUCTION

During the rut, North American bison (Bovidae: *Bison bison*) bulls are known to engage in head-to-head pushing, shoving, and hitting to both assert their dominance and to win the right to mate with a bison cow (Fuller, 1960; Lott, 1974, 2002; McHugh, 1958). Recent research focused on the brains of male bighorn sheep (*Ovis canadensis*) and male muskoxen (*Ovis moschatus*) – bovids that also engage in head-to-head impacts – which suggests that these animals may be susceptible to acute or chronic traumatic brain injury (TBI) (Ackermans et al., 2022). Muskoxen may be particularly susceptible to TBI as phosphorylated tau protein, a hallmark of neurodegenerative disease, was found in the sulci and around the blood vessels of muskoxen, while less tau accumulation was found in the brains of bighorn sheep. The impacts incurred by bighorn sheep are dissipated and attenuated by their horns (Johnson et al., 2017), but the muskoxen impacts occur along the horn boss, a bony structure spanning the forehead from horn to horn (Alaska Department of Fish and Game, 2024), and how energy is dissipated along this structure remains unclear. Bison also impact one another along the forehead, but bison lack a horn boss, and therefore receive blunt impacts directly to the skull with only the hair and scalp acting as a buffer, which suggests that the impact energy that is not dissipated by the hair and scalp is transmitted directly to the skull. Ackermans et al. (2022) have suggested that the thickness of the male bovine skull may provide some protection from brain injury. While the social behaviors of North American bison have been documented in detail by McHugh (1958), little research has focused on the cranial anatomy of the bison and how the cranial anatomy dissipates the impact energy when two bulls collide.

**Fig. 1.**
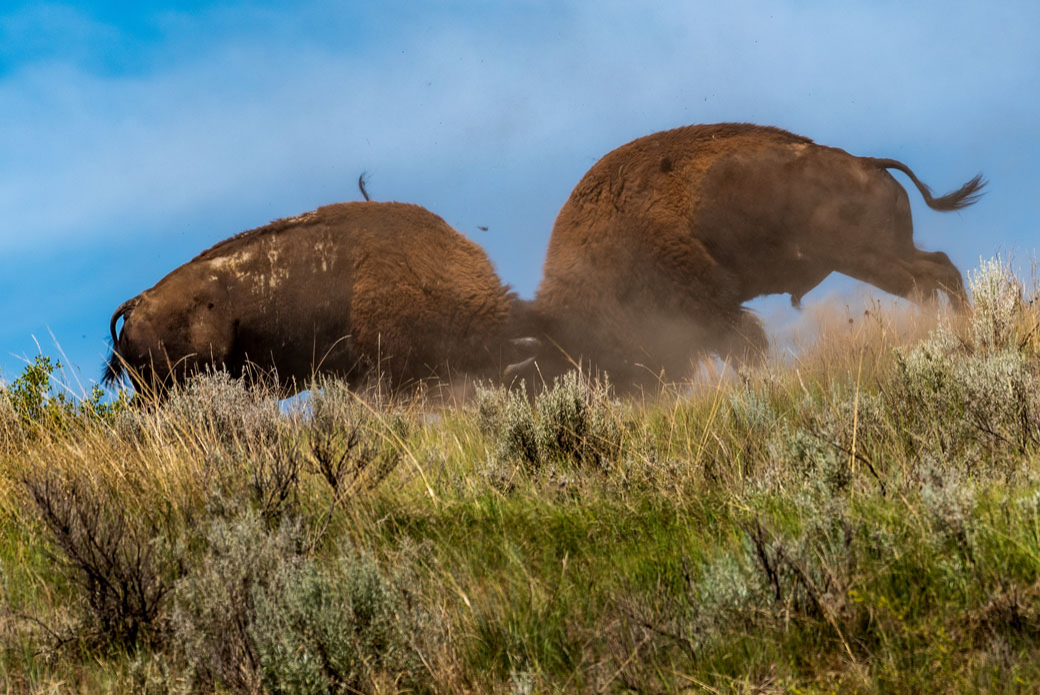
**North American bison bulls engaging in head-to-head fighting.** Photo by Randy Runtsch (stock.adobe.com).

**Fig. 2.**
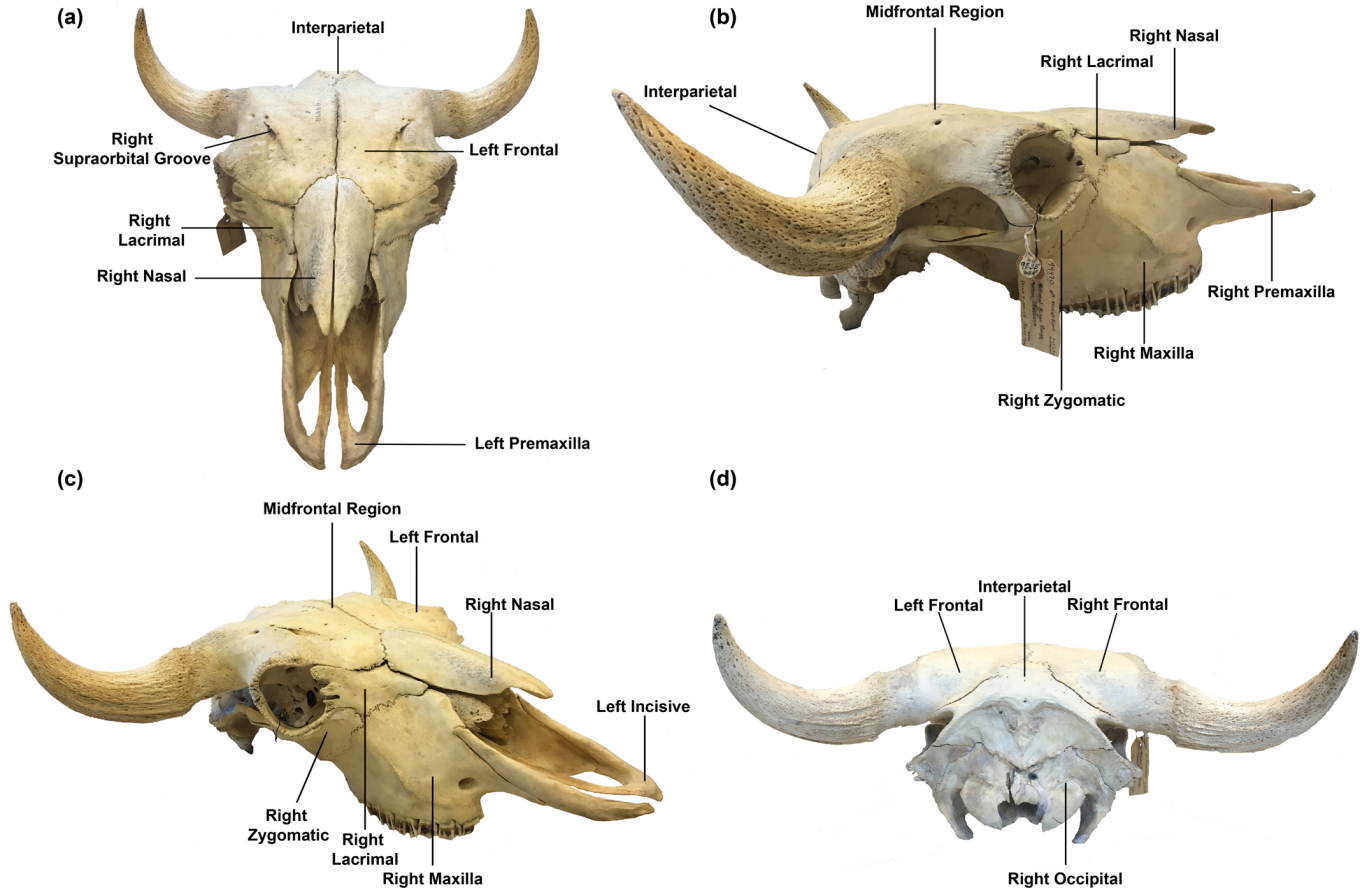
**External anatomy of the North American bison skull.** The interparietal bone is the location of the majority of impacts. (A) Cranio-dorsal view of the skull. With the exception of the interparietal bone, the external bones are paired (left and right). (B) Lateral view of the skull. Note the raised nature of the midfrontal region. (C) Off-axis view of the skull. (D) Caudal view of the skull. Note the location of the interparietal bone.

Fighting between bison begins either with one bull slowly approaching the other, with one bull shaking his head at the other, or with one bull charging the other (Fuller, 1960; Lott, 1974, 2002; McHugh, 1958). While the bulls may attempt to use their horns to gore the flank of their opponents, the main fighting mechanism consists of head-to-head ramming about the interparietal bone (Figs 1 and 2) and the frontal bones (Kreutzer, 1992; Soana et al., 1996), followed by head-to-head shoving. In this situation, the dominant bull may push his opponent backwards by several tens of feet and in some cases, cause the opponent to be flipped onto his back (Fuller, 1960; Lott, 1974, 2002; McHugh, 1958).

Similar to the domestic cow, the outer skull of the bison comprises paired symmetric premaxilla, nasal, frontal, maxilla, lacrimal, and zygomatic bones and the singular interparietal bone (Budras et al., 2011; Liebich and König, 2014) (Fig. 1A-D). The external anatomy of the bison skull, however, provides little insight into how the frontal and interparietal bones dissipate the energy produced during head-to-head contact, suggesting that an underlying bony support structure that works with the frontal and interparietal bones to dissipate energy may be present. Based on developmental biological studies of the fetal bovine skull, ossification of the frontal bones occurs between days 45 and 52 of gestation, and by day 97, the substantia corticalis, a thin region of cortical bone underlain by thin trabeculae that help distribute dynamic pressures, has formed (Matthews, 1972; Soana et al., 1996). As the fetus continues to develop, the nasal mucosa inverts into the trabeculae underlying the frontal bones to form the paranasal sinuses. After birth, the paranasal sinuses continue to develop and eventually extend to underlie the frontal and interparietal bones (Budras et al., 2011). The paranasal sinuses are separated by septa formed from bone and membranous tissue. These septa vary not only in their geometries, sizes, and angles within an individual bison but are also variable when comparing one bison to another (Budras et al., 2011; Farke, 2010) (Fig. 2B).

Three main structural bone types have been recognized in bovid skeletons and include: (i) cortical or haversian bone, (ii) cancellous or trabecular bone, and (iii) plexiform or fibrolamellar bone. Additionally, a combination of cortical and plexiform bone has also been observed in North American bison and domestic cattle (Burr, 2019; Currey, 2003; Locke, 2004; Martin and Boardman, 1993; Novitskaya et al., 2011).

Cortical bone is a dense, low-porosity bone comprised of osteons oriented along the longitudinal axis of the bone. Each osteon contains a central haversian canal encompassed by concentric lamellae and is differentiated from adjacent osteons by the presence of a cement line. The individual osteons are, however, linked by Volkmann's canals that help in bone perfusion (Thompson, 2016). Conversely, cancellous bone comprises a highly porous lattice of plate- and rod-shaped trabeculae that orient along the principal stress axis of each bone. The open structure of cancellous bone allows for infilling with marrow bearing hematopoietic cells (Burr, 2019; Thompson, 2016). Plexiform bone, which contains interconnected vascular plexuses, is typically found in large, rapidly growing animals and comprises lamellar bone underlain by a core of woven bone, creating a brick and mortar-type appearance (Burr, 2019; Newman et al., 1995; Pearce et al., 2007).

The mechanical properties also vary among bone types because of multiscale porosity levels associated with each type of bone. Cortical bone behaves as a transversely isotropic material where the mechanical properties are the same in a plane that lies perpendicular to the longitudinal axis of the bone. Further, while cortical bone is stiffer under axial loading (Barrera et al., 2016), the strength of cortical bone is affected along the longitudinal axis by porosity, but the porosity does not affect the strength in the transverse plane (Dong and Guo, 2004). With mechanical properties that vary in different directions, cancellous bone is anisotropic in nature, while plexiform bone behaves as an orthotropic material, having mechanical properties that are specific to its axial, transverse, and radial planes (Barrera et al., 2016; Katz and Yoon, 1984; Keaveny et al., 2001). Plexiform bone is stiffer than cortical, especially under axial loading, and depending on its anatomical location within the bone, may have a higher elastic modulus. Further, plexiform bone tends to have a higher porosity than that of cortical bone due to the numerous vascular plexuses (Barrera et al., 2016; Katz and Yoon, 1984; Martin and Boardman, 1993) (Table 1).

**Table 1.**
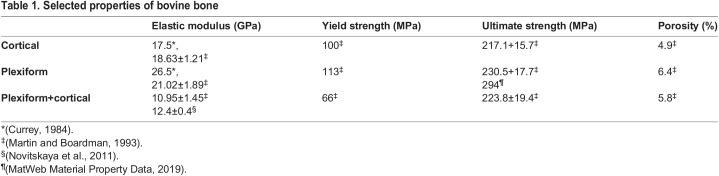
Selected properties of bovine bone

A three-dimensional finite element model was used to explore the material properties and behavior of the interparietal bones, frontal bones, and bony septa within the parasagittal sinuses under head-to-head loading conditions encountered during the rutting season. As quantitative data regarding the velocity of the bison during head-to-head contact are lacking, video analysis of bison bulls fighting during the rut was used to determine the theoretical boundary conditions for the finite element (FE) model. A range of potential velocities at which bison may collide including low velocity, mild impacts (2235.2 mm/s), mid-velocity, moderate impacts (4470.4 mm/s), and high velocity, extreme impacts (6705.6 mm/s) were incorporated into the model. Further, three theoretical impact scenarios were modeled including direct contact between the interparietal bones and direct contact between the midfrontal bones in the sagittal plane, and an oblique hit where the interparietal bone of one bison in the parasagittal plane impacted the midfrontal bone of the other bison in the sagittal plane. The theoretical boundary conditions were then used to explore the interaction between stress and strain at each velocity and impact scenario to determine how this interaction was absorbed and dispersed throughout the bison skull.

## RESULTS

### Mises stress contours

The Mises stress contours for the interparietal, midfrontal, and oblique impacts are shown in Fig. 5. When bison headbutt, the impacts typically occur along the interparietal bone. In the FEA skull, the average thickness of the interparietal was measured as 19.6 mm. At the lowest impact velocity (2235.2 mm/s), the highest stresses of 10-50 MPa concentrated along the bony septa underlying the interparietal bone and within the interior of the skull, while lower stresses of 1-10 MPa distributed throughout the interior of the skull and along the curvature of the braincase. When the velocity of the interparietal impact increased to 4470.4 mm/s, most of the stress along the bony septa and the interior of the skull ranged still ranged from 10-50 MPa; however, localized regions of high stress ranged from 10-50 MPa along the curvature of the braincase. At the greatest interparietal impact velocity of 6705.6 mm/s, the stresses for predominantly ranged from 10-50 MPa, with localized stresses between 150-294 MPa occurring along the bony septa and interior of the skull underlying the interparietal bone (Fig. 5).

**Fig. 3.**
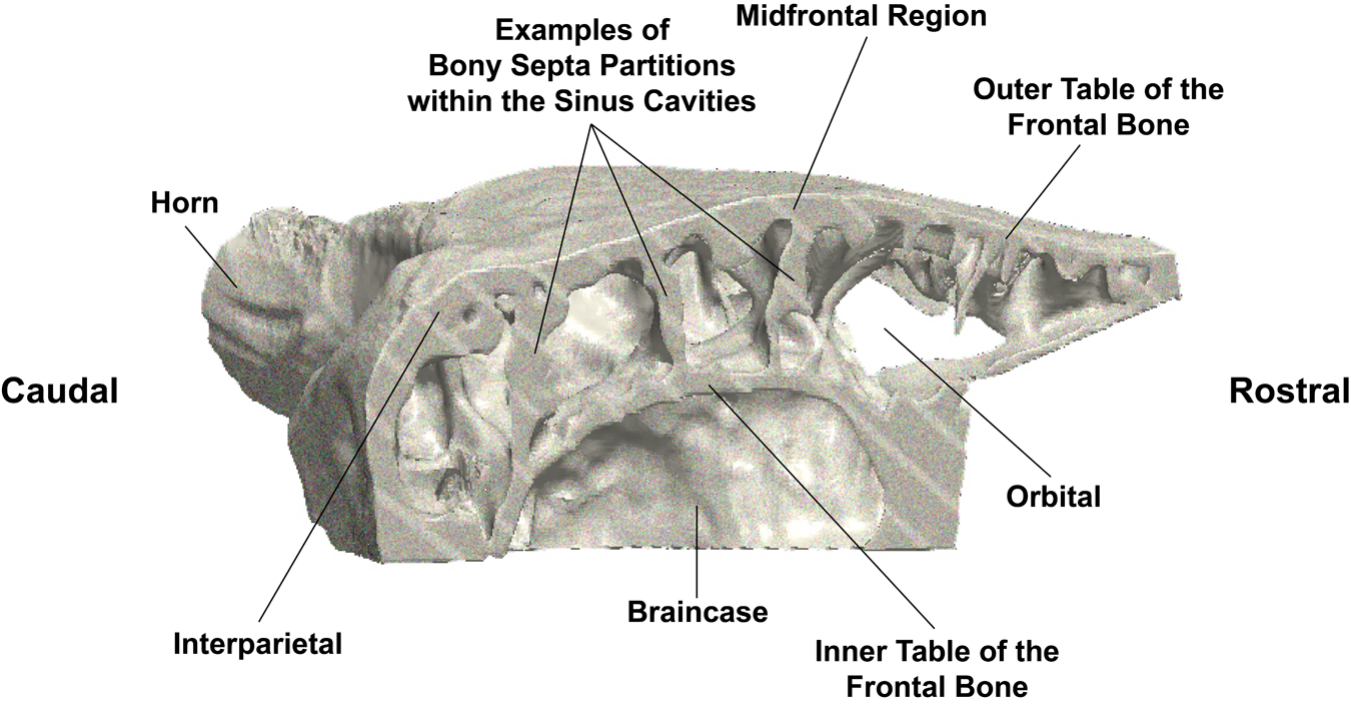
**Three-dimensional image of rendered from a CT scan of the internal parasagittal cross-sectional anatomy of a North American bison skull.** Note the thickness of the interparietal bone (Budras et al., 2011).

**Fig. 4.**
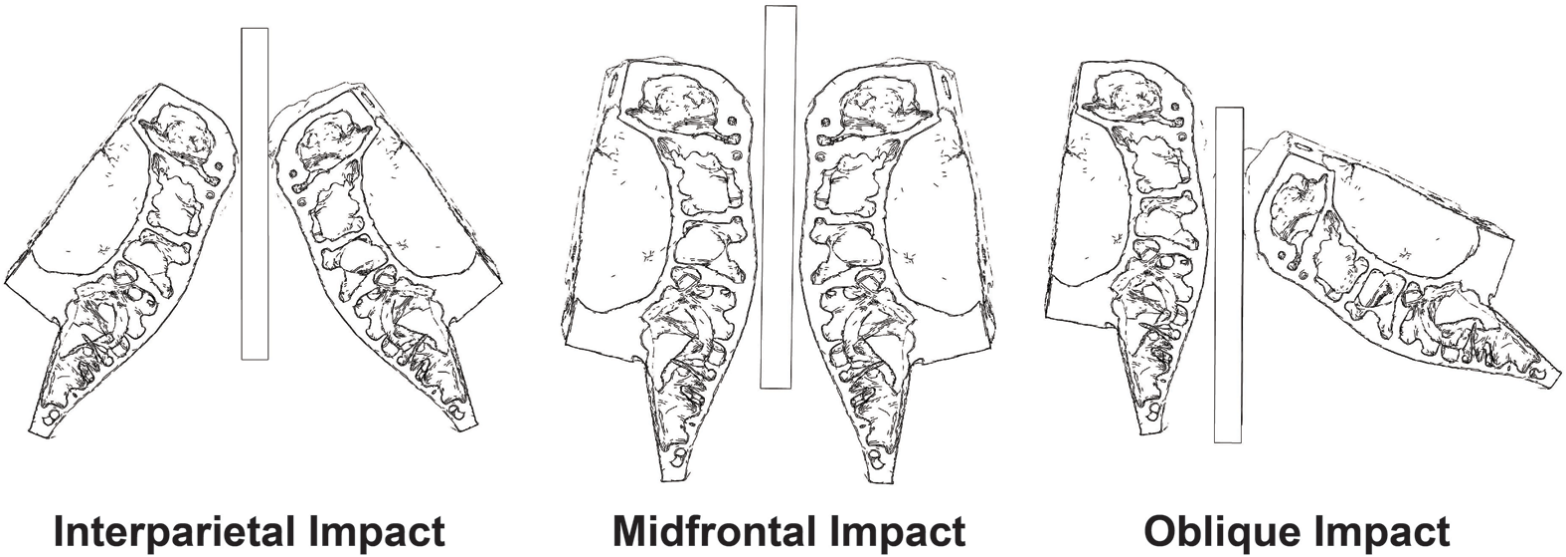
**Examples of the simulated boundary conditions.** Interparietal impacts consisted of direct impacts to the interparietal bones of each skull. Midfrontal impacts consisted of direct impacts between the midfrontal regions of each skull, while oblique impacts consisted of impacts between the interparietal bone of the right skull and the midfrontal region of the left skull.

**Fig. 5.**
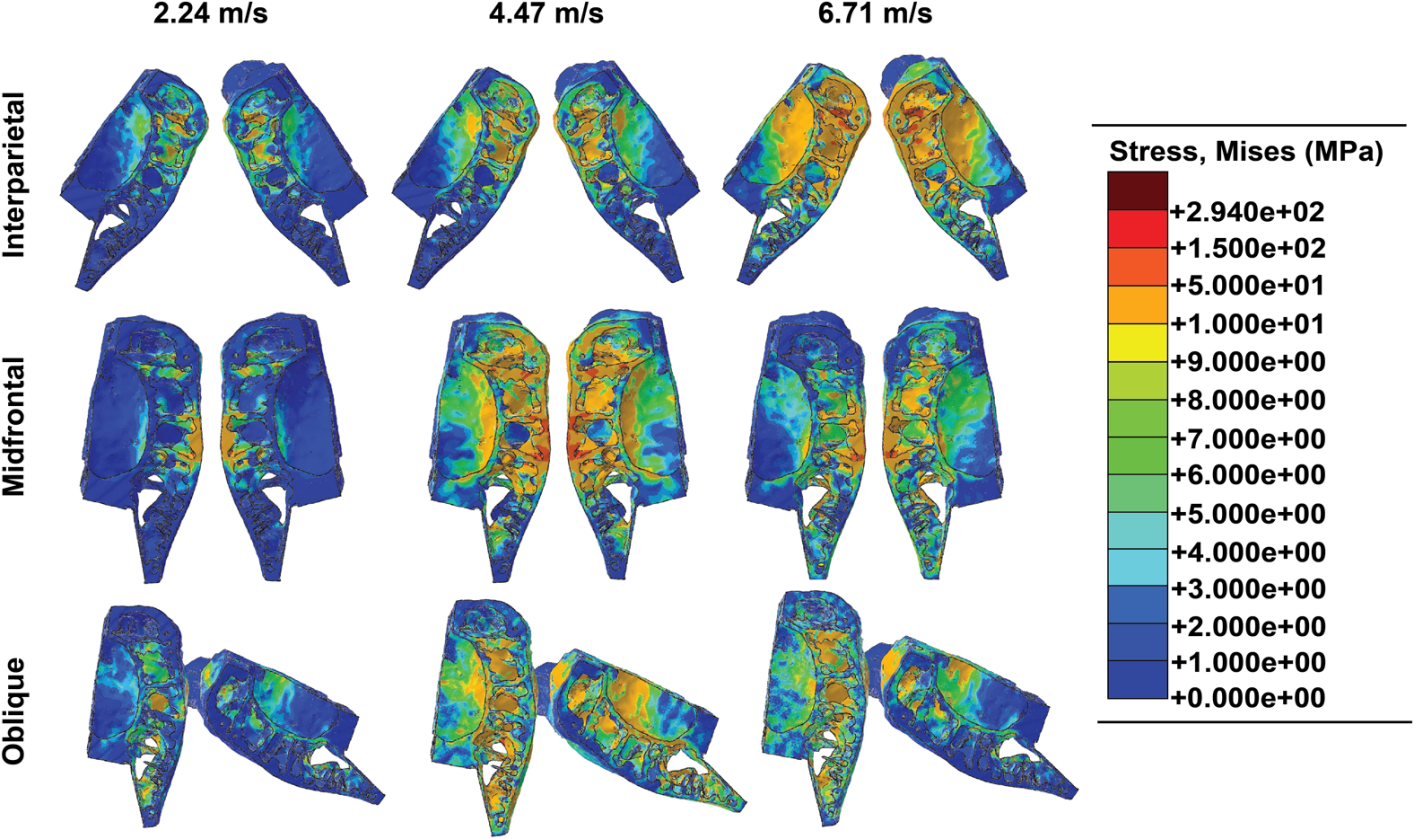
**Von Mises stress contours for each impact location by each velocity.** As the velocity increased, the stresses concomitantly increased. Localized stresses were higher in midfrontal impacts. The global stresses produced in each simulation did not exceed the compressive strength of plexiform bone (294 MPa). The ballistic gel was removed from the output to facilitate viewing of the stress contours.

The stresses that occurred following midfrontal impacts resulted in localized regions of high stress. Impacts occurring at 2235.2 mm/s produced stresses ranging from 10-50 MPa along the impact location, which, based on the FEA, were not transmitted into the braincase. Small, localized stress concentrations of 150-294 MPa occurred at the impact location and in the skull interior. At a velocity of 4470.4 mm/s, the stresses on the skull ranged between 10-50 MPa, but these stresses were transmitted into, and follow the curvature of, the braincase. Localized stress concentrations between 150-294 MPa were found primarily at the impact location. Midfrontal impacts at the highest velocity (6705.6 mm/s) produced stresses between 10-50 MPa at the impact location, but some regions of localized stress ranging from 150-294 MPa also occurred at the impact location. Stresses between 10-50 MPa were carried into the braincase but only occurred directly underneath the portion of the frontal bone receiving the direct impact. At neither 2235.2 mm/s nor 4470.4 mm/s did the 10-50 MPa stress levels extend into the tip of the frontal bone. However, at 6705.6 mm/s the 10-50 MPa stress levels did reach to the frontal bone tip (Fig. 5).

The oblique impacts produced slightly greater stress levels than those of midfrontal impacts at the midfrontal bone although the other regions of the skull exhibited similar stress levels. Stresses from 10-50 MPa were present throughout the impacted midfrontal skull at all velocities (Fig. 5). At a velocity of 4470.4 mm/s, localized strains and the associated stresses in the range of 150-294 MPa were visible along the interior septa of the skull impacted along the midfrontal region. In contrast, the skull impacted along the interparietal bone experienced stresses between 10-50 MPa that occurred over a smaller region than that of the midfrontal bone.

### Maximum principal strain contours

**Fig. 6.**
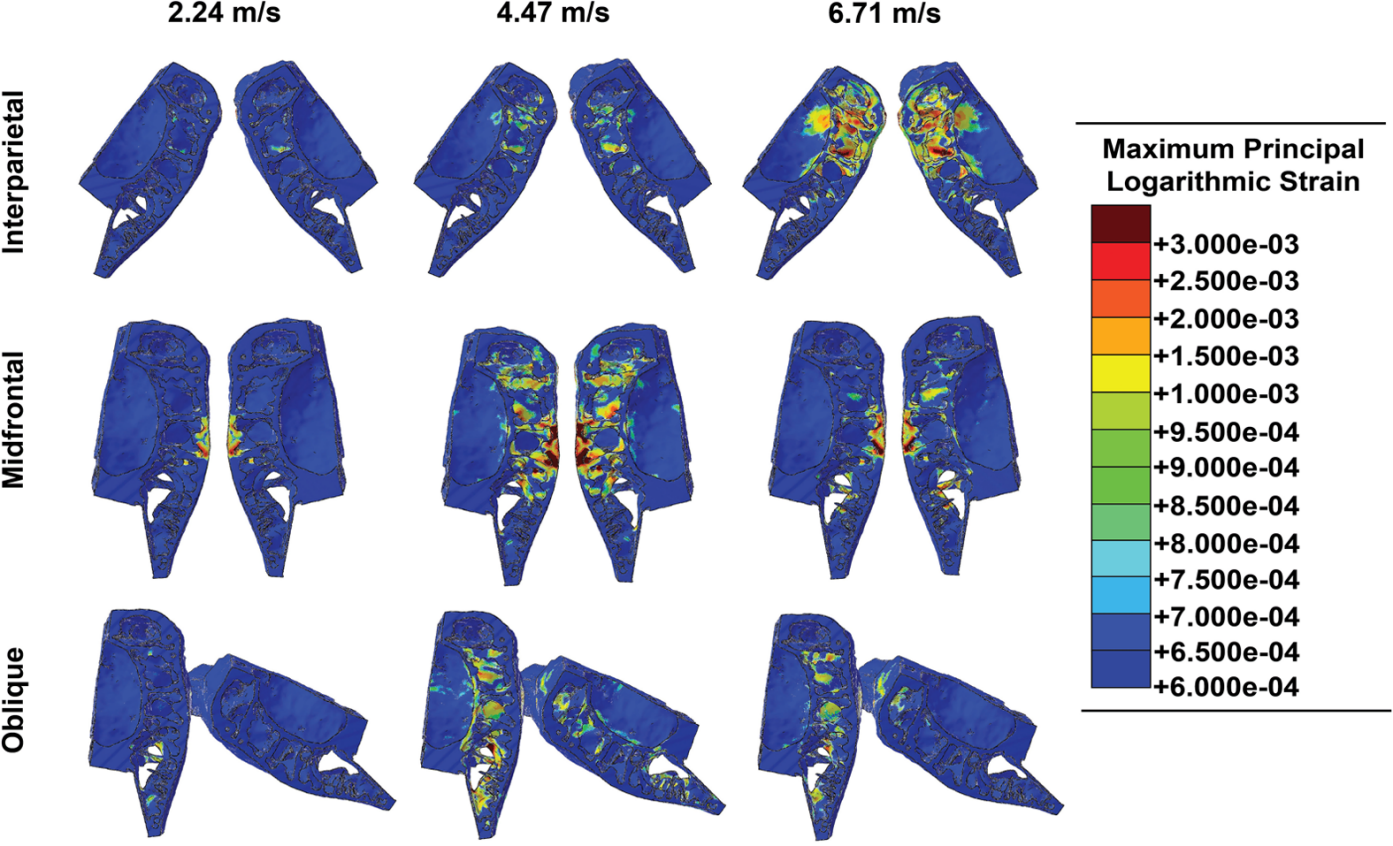
**Maximum principal strain contours for each impact location by each velocity.** Similar to the stress contours, midfrontal impacts produced greater focal strains. Overall, the strain waves produced by oblique impacts were the greatest and were dispersed throughout the skull. None of the simulations produced strains in excess of the yield strain. The ballistic gel was removed from the output to facilitate viewing of the strain contours. Note: strain is dimensionless.

Strain contours resulting from the interparietal, midfrontal, and oblique impacts are depicted in Fig. 6. Interparietal impacts occurring at a velocity of 2235.2 mm/s produced small regions of strain ≤0.0015 within the interior of the skull. At a velocity of 4470.4 mm/s, the strain was still mostly confined to the interior of the skull, with a localized region of strain ranging between 0.002 and 0.0025. Impacts occurring at the highest velocity of 6705.6 mm/s produced strains throughout the interparietal region ≤0.003; however, a small interior region of the skull reached strain levels in excess of 0.003.

The strains produced during midfrontal impacts remained localized at the area of impact. Strain intensities were similar to those produced during interparietal impacts, regardless of impact velocity. At the impact velocities of 4470.4 mm/s and 6705.6 mm/s, local strains ≥0.003 were produced.

The strain intensities produced by oblique impacts were also similar to those produced by interparietal and midfrontal impacts; however, the strain was more pronounced in the skull impacted about the midfrontal region, with localized regions reaching strains ≥0.003. Strains in the skull impacted along the interparietal bone were lower and ranged from 0.0007-0.0015.

**Fig. 7.**
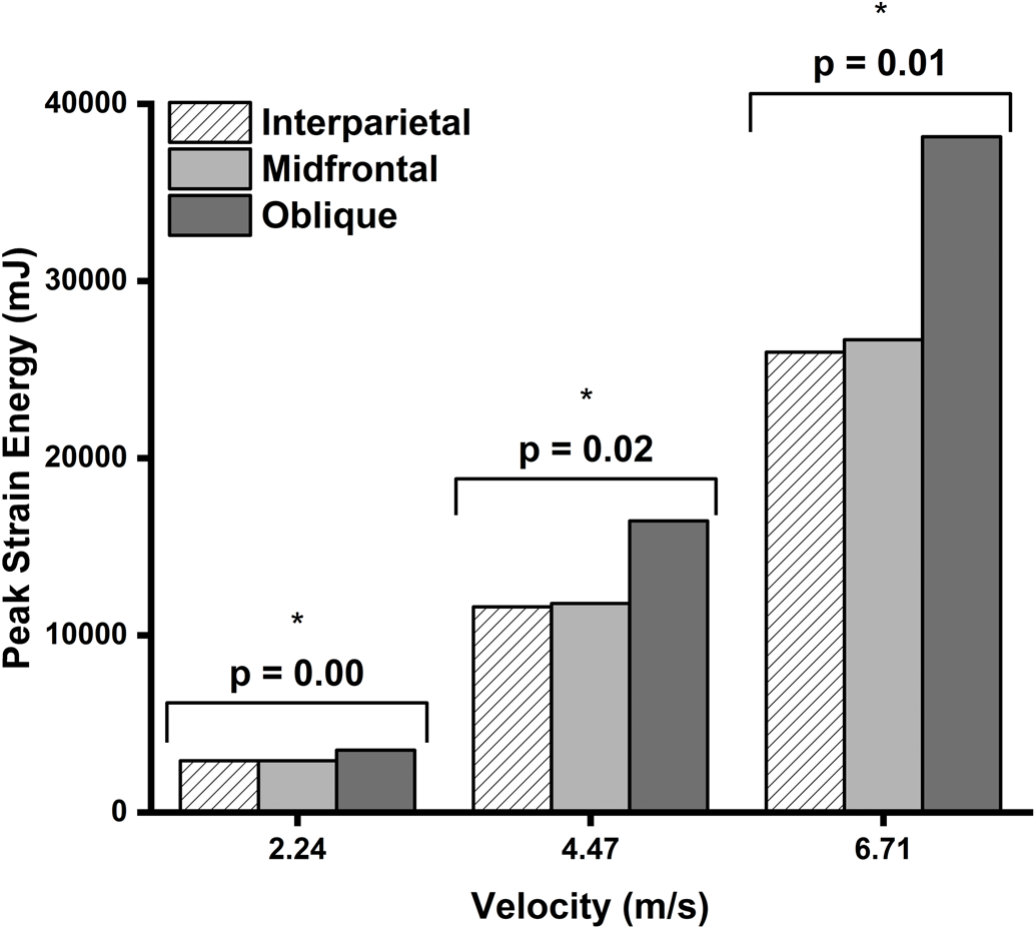
**The peak strain energy produced at each impact location for each velocity.** A Shapiro-Wilk test (α=0.05) was used to test for normality of the data. The data were normally distributed and based on the results of a two-sided *t*-test (α=0.05) oblique impacts produced statistically significantly higher strain energies at all velocities (2.24 m/s, *P*=0.00; 4.47 m/s, *P*=0.02, and 6.72 m/s, *P*=0.01) than those produced by contact between the interparietal bones, which is the natural site of impact during headbutting.

Fig. 7 shows the comparison of the three impact velocities for the three impact scenarios. The interparietal and midfrontal impacts gave essentially the same peak strain energy; however, the oblique impact gave the highest statistically significant peak strain energy produced in the simulations. The oblique impact linearly increases its percentage of its admissible peak strain energy as the impact velocity increases and is statistically significant at all velocities simulated: 2235.2 mm/s (*P*=0.00), 4470.4 mm/s (*P*=0.02), and 6705.6 mm/s (*P*=0.01).

## DISCUSSION

Two overall patterns are evident from the FE simulations. First, as the velocity increased, the magnitude of the propagating stress and strain waves increased, extending globally throughout the skull. As the stresses dynamically progressed to greater volumes, the magnitudes of the stresses remained below the fracture threshold of 294 MPa for plexiform bone (Novitskaya et al., 2011) throughout every simulation. Second, interparietal impacts produced the lowest stresses and strains with respect to the impact velocities globally because the interparietal bone spread out the loads; however, oblique impacts resulted in the largest stresses and strains with respect to the impact velocities especially in the midfrontal skull region (Figs 5 and 6).

**Fig. 8.**
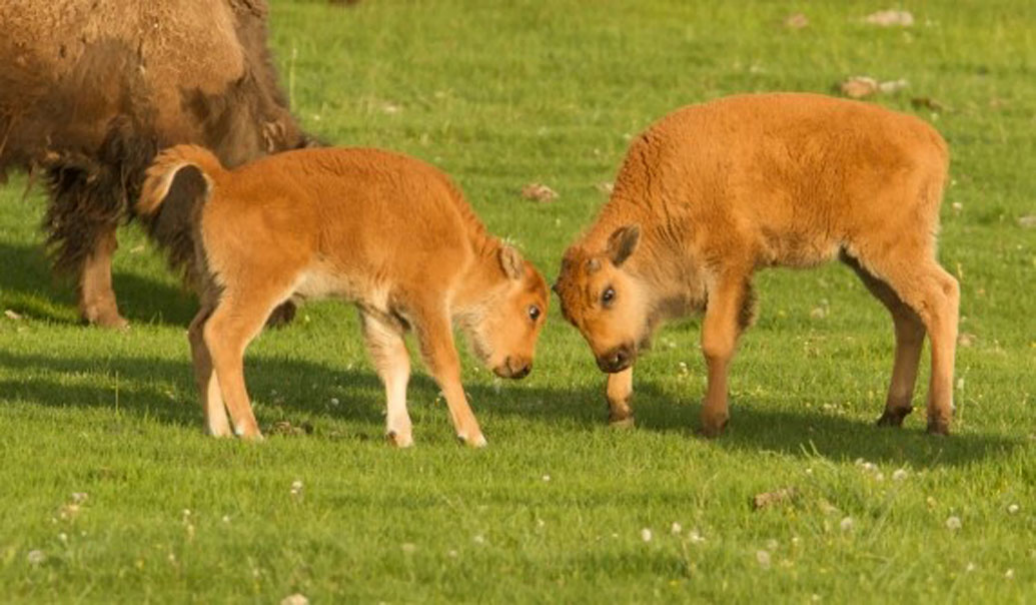
**North American bison calves engaging in “play fighting” behavior.** Photo taken by, and used with the permission of, Mike Dunn.

With respect to the energy absorption, regardless of the impact location, the peak strain energy increased as the impact velocity increased (Fig. 6) as expected. Impacts along the interparietal bones, of which naturally occur when bison engage in head-to-head fighting (Kreutzer, 1992), produced the lowest peak strain energies, while oblique impacts produced statistically significantly higher peak strain energies (Figs 6 and 7). The initial loads of the direct head-to-head impact are larger than oblique ones and occur more often than oblique ones. These findings suggest that the interparietal bone is designed to take the impacts resulting from head-to-head fighting. As mentioned, the bison interparietal bone is thicker than the adjacent bones, which makes it more efficient at absorbing and distributing the energy during an impact. Whether the interparietal bone is thicker than the other bones at birth is unknown. Bison calves are known to “play fight” (Fig. 8), and the possibility exists that the interparietal bone conforms to Wolff's Law and increases in thickness with each impact incurred from “play fights” through reproductive maturity (Wolff, 1986).

Oblique impacts, whereby the thickened interparietal bone of one bison strikes the thinner frontal bone of the other, produced the greatest peak strain energies, but bison are not normally expected to engage in this type of impact. These boundary conditions and the design to optimize the weight and strength of the bones, allows both the stress and strain energies to increase and globalize about the skull. Also, strain intensities in the skull impacted along the midfrontal bone tended to be greater than those produced in the skull impacted along the interparietal bone, lending further support to the hypotheses that the thicker interparietal bone was more efficient in absorbing and distributing impact energies.

While the peak strain energies produced in midfrontal impacts were lower than those produced in oblique impacts, midfrontal impacts also created localized stress and strain concentrations along the septa directly underneath the impact locations (Figs 5 and 6). Further, as there was little to no angle between the skulls during midfrontal impacts, more area was in contact during the impact; however, as the midfrontal bones were of relatively the same thickness, the peak strain energies were not as high as those produced during oblique impacts.

The results of these analyses suggest that the function of the bony septa that underlies the interparietal and frontal bones help with impact mitigation. Theoretically, an increase in thickness should concomitantly increase the impact absorption capabilities of the interparietal bone while decreasing its susceptibility to bending. Overall, the patterns of the stress contours and principal strains suggest the fracture would not occur in most cases for bison skull bones; however, if a bison bull were to engage in the maximum number of fights with maximum impact velocities in each rutting season of reproductive viability, damage of the bone could occur.

Several limitations of this study should be noted and can be examined in future studies. Due to a lack of information regarding the material properties of the bison brain, neither a brain nor the cerebrospinal/sinus fluids were included in the FE models reported here, creating difficulties in determining if the stress wave and strain energy produced during an impact is transmitted to the brain, and how the energy affects the brain. Future studies that examine the bison brain for signs and locations of TBI could further inform the model to determine how effective the interparietal bone and bony septae are in dissipating the energy produced when two bulls collide. Further, in the FEA model, only linear elastic material properties were incorporated, whereas bone exhibits a complicated nonlinear viscoelastic behavior. The viscoelastic model should dissipate more energy than an elastic material, and if used in the FEA analysis would have given lower stress values than are shown herein. Future studies should also consider incorporating the mass of the bison. Additional consideration of the microstructural characteristics of the combined plexiform and haversian (cortical) bone found in the bison skull could further inform the choice of material model. Also, a conservative thickness of 16 mm was applied to the ballistic gel, but recent research suggests that in mature bison bulls the hide overlying the frontal bone may be as thick as 38 mm (Rioja-Lang et al., 2019). Finally, the atlas and axis of the vertebral column of the bison are relatively dense and have been hypothesized to act as shock absorbers (Kreutzer, 1992), but the current model does not include these vertebral components or their associated musculature. Future models that overcome these limitations will not only provide insights into North American bison ethology and anatomy but will also contribute to the understanding of natural systems of impact mitigation and how effective these systems are in mitigating TBI.

## MATERIALS AND METHODS

### Bison skull model development and mesh

The skull of an adult male bison bull recovered from the National Bison Range in Montana was used in simulations to assess of the impact mitigation capabilities of the North American bison skull. The bison skull was loaned to the authors by the Museum of Vertebrate Zoology at the University of California, Berkeley (Accession Number Mamm MVZ 99970). Externally, the skull was intact with no fractures, but the lower mandible was not included. The total length of the skull (tip of the premaxilla – mid-interparietal) was approximately 526.4 mm, while the maximum width of the skull (outer right orbital – outer left orbital) was approximately 331.2 mm for a theoretical impact area of 174,343.68 mm^2^. Skinner and Kaisen (1947) measured 29 different North American bison and measured lower and upper bounds on the length of skulls between 491 and 570 mm, respectively, with a mean value of 541 mm. Furthermore, Skinner and Kaisen (1947) measured the widths ranging from 271 to 343 mm, with a mean value of 317 mm revealing a difference of 4.4% when compared to the bison skull analyzed in this study (Skinner and Kaisen, 1947). Following photographing and measuring the external features of the skull, the internal features were accessed through computed tomography (CT) scanning of the skull. The digital images and communication in medicine (DICOM) files produced by CT scans of the skull were imported into Simpleware™ ScanIP (N-2018.03-SP2 Build 55) and used to render a three-dimensional model of the entire skull resulting in 5,186,280 voxels.

To further facilitate the viewing of internal anatomy of the frontal and interparietal bones, to decrease the computational time of the simulations, and to prevent the presence of islands of isolated voxels that would compromise the simulations, the skull was then halved approximately along the interfrontal suture to create a symmetry plane. Also, the lower portion of the braincase was removed. Additionally, the nasal and premaxilla bones and their associated structures including the upper teeth and diffusive nasal tissue were removed. The frontal and interparietal bones had an average thickness of 8.9 mm and 19.6 mm, respectively. The thickness of the frontal bone varied along its length and was thickest at the middle (midfrontal). Within the midfrontal region, an average thickness of 14.6 mm was recorded. The average inner distance between the outer and inner tables of the frontal bone was approximately 33.0 mm. Both the outer table of the frontal bone and the interparietal bone are underlain by the paranasal sinuses that are separated by bony septa of various geometries and sizes. Some septa connect the outer table of the frontal bone to its inner table. The paranasal sinuses overlie the braincase (Figs 1B and 2). These dimensions are key for the stress and wave analyses, which in turn play a role in determining the mechanisms for wave dispersion and energy absorption. The reduced model was then meshed in Simpleware™ ScanIP (N-2018.03-SP2 Build 55) using the coarsest meshing option to produce a quadratic tetrahedral mesh that was subsequently imported into Abaqus 2017 (Dassault Systèmes). The final mesh for the skull consisted of 312,726 tetrahedral quadratic (C3D10 M) elements with an associated 534,731 nodes.

### Material model

Using the imported meshed skull, the explicit finite element method was implemented to simulate bison head-to-head impacts. First, the imported meshed skull was mirrored as a plane of symmetry to create two bison skulls, and a 16 mm thick layer of linear elastic ballistic gel meshed in Abaqus 2017 (Dassault Systèmes) was placed between the two skulls to approximate the scalp and hair (Rioja-Lang et al., 2019). The gel comprised 30,000 linear hexahedral (C3D8R) elements with an associated 36,057 nodes.

A prior histological analysis of the interparietal bone, frontal bone, and the bony septa identified the presence of a mixture of haversian (cortical) and plexiform bone (Beresford, 1993; Persons, 2019). The elastic modulus of cortical bone ranges from 17.5 GPa (Currey, 1984) to 18.63 GPa (Martin and Boardman, 1993). The elastic material properties taken from the literature used a mixed bone type that was found in bovines and included a modulus of 12,400 MPa (Novitskaya et al., 2011), a density of 2.06×10^−9^ tonnes/mm^3^ (1868.8 kg/m^3^) (Martin and Boardman, 1993; Novitskaya et al., 2011), and a Poisson's ratio of 0.34 (Van Buskirk et al., 1981). The material properties assigned to the elastic ballistic gel included a modulus of 210 MPa (Al Khalil et al., 2019), a density of 1.25×10^−9^ tonnes/mm^3^ (1133.9 kg/m^3^) (Datoc, 2010), and a Poisson's ratio of 0.3 (Al Khalil et al., 2019). Based on the compressive strength of plexiform bone, an upper limit of 294 MPa was used as the failure criterion for the model.

Although the modulus of bone varies based on its type and location, a single modulus was applied to all bone in the FEA model. The histology of the interparietal and frontal bones was found to be a combination of haversian (cortical) and plexiform bone, and all samples exhibited similar interspersion of the bone types (Persons, 2019).

Outcome measures for the FEA results were focused on determining the patterns of energy transmission throughout the skull by examining: (i) the Mises stress (von Mises, 1913), which mathematically considers all of the components of the stress tensor, and (ii) the principal strain levels within the skull in order to quantify if fracture could occur.

### Boundary conditions

An initial velocity was applied to each skull (equal and opposite in value) to simulate head-to-head impacts. Because movement of the bulls was not constrained by locked horns during headbutting, neither the ballistic gel separating the skulls nor the skulls themselves were encastred (fixed in place), allowing for displacement in any direction. To estimate the velocity of a bison prior to impact, a video analysis of a stock video from National Geographic (National Geographic, 2011) of bison fighting was performed in Kinovea version 0.8.15 (www.kinovea.org). Video markers were placed on the head of the bison, and the video was stepped frame-by-frame until the frame in which the impact occurred was reached, and the impact velocity was recorded. The head velocities of the bison ranged from 0.46 m/s to 14.15 m/s with a mean velocity of 8.65 m/s. Based on the video analysis, three different velocities were applied across three impact scenarios. The applied velocities were 2.24 m/s (2235.2 mm/s), 4.47 m/s (4470.4 mm/s), and 6.71 m/s (6705.6 mm/s). Fig. 5 shows the three theoretical impact scenarios implemented in the model: (i) contact between the interparietal bones of both skulls (interparietal) to simulate the natural loading conditions similar to Fig. 1, (ii) contact between the midfrontal regions of both skulls (midfrontal), and (iii) contact between the interparietal bone of the right skull and the midfrontal region of the left skull (oblique) (Fig. 4).
